# Usefulness of intraoperative C-arm image intensifier in reducing errors of acetabular component during primary total hip arthroplasty: an application of Widmer’s method

**DOI:** 10.1186/s12891-021-04791-8

**Published:** 2021-10-20

**Authors:** Joo-Hyoun Song, Yong-Sik Kim, Soon-Yong Kwon, Young-Wook Lim, Jiyoung Jung, Seungbae Oh

**Affiliations:** 1 Department of Orthopaedic Surgery, St. Vincent’s Hospital, The Catholic University of Korea, Suwon-si, Gyeonggi-do Republic of Korea; 2 Department of Orthopaedic Surgery, Seoul St. Mary’s Hospital, Seoul, Republic of Korea; 3 Department of Orthopaedic Surgery, Eunpyeong St. Mary’s Hospital, Seoul, Republic of Korea; 4 Department of Orthopaedic Surgery, College of Medicine, The Catholic University of Korea, Seoul, Republic of Korea

**Keywords:** X-rays, Hip, Arthroplasty, Replacement, Acetabulum

## Abstract

**Background:**

Acetabular prosthesis positioning in total hip arthroplasty (THA) is crucial in reducing the risk of dislocation. There has been minimal research on the proper way to put the acetabular components into the safe zone intraoperatively. Assessment of version by intraoperative imaging intensifier is very valuable. The value of Widmer’s method, using the intraoperative C-arm available to determine cup anteversion was assessed.

**Methods:**

One hundred one hips in 91 patients who underwent primary THA were eligible for inclusion. Utilizing intraoperative C-arm images, measurement was performed using the technique described by Widmer. The values obtained using 3D computed tomography postoperatively, which determined the anteversion of the acetabular component, were regarded as the reference standard.

**Results:**

The method of Widmer obtained values similar to those obtained using 3D computed tomography and was considered accurate (n.s.). All 101 hips were positioned in the set target zone. Among the 101 hips, the cup position in nine hips (8.9%) was changed. The dislocation rate in our study was 1.0% with all dislocations occurring in hips placed in the target zone. The mean Harris hip score after THA in 1 year was 94.2 (82-98).

**Conclusions:**

The method of Widmer was accurate using intraoperative imaging intensifier for the measurement of the anteversion of the acetabular component during THA, with reference to the anteversion obtained from the 3D computed tomography. Also, utilizing intraoperative C-arm imaging was very useful because it allowed for correction of the position of the acetabular cup.

## Background

In the field of total hip arthroplasty (THA), ideal location of the acetabular component cannot be overly overemphasized for the ultimate success of the surgery. Acetabular component malposition has been associated with impingement, subluxation or dislocation, increased wear and osteolysis [1, 2].

The orientation of the acetabular component in THA is defined by inclination, which is the angle between the face of the implant and the transverse interteardrop axis; and version, which is the angle between the axis of the component and the coronal plane of the patient [3]. Lewinnek et al. proposed a ‘safe zone’ of cup inclination of 40 ± 10 and anteversion of 15 ± 10 to minimize dislocation risk after primary THA [4] and has been regarded as acetabular component position standards. However, dislocation has still been reported even though the cup has been in the safe zone [5].

Inclination can easily be measured on anteroposterior (AP) radiographs relatively. However, version is more difficult to measure. There are a variety of methods of measuring the version on plain AP or cross-table lateral radiographs. Six methods (Lewinnek; Widmer; Hassan, et al.; Ackland, Bourne and Uhthoff; Liaw, et al.; and Woo and Morrey) exist [4, 6–10]. However, reliability and validity of these six methods are known to be different. Validity of the methods of Widmer and of Akland, Bourne and Uhthoff were questioned by some [11]. Conversely, there were other reports that Widmer’s method is the reliable way for evaluating the anteversion of the acetabular component on plain radiographs [12].

The version of the acetabular component can be measured more accurately using CT scans what could be demonstrated in previous studies [13]. However, plain radiographs are more frequently used clinically since CT scans incur additional radiation and are costly. To date, few alternatives other than the navigation system have been proposed to measure version exactly [14].

Few attempts have been made to measure and correct the anteversion of the cup during surgery using any of the methods above. Despite the widespread availability of standard X-ray equipment, the use of intraoperative X-rays for THA has rarely been evaluated. Though some studies have evaluated intraoperative radiographs for checking leg lengths discrepancy during total hip arthroplasty, the utility of such radiographs to guide component orientation has not been formally evaluated [15, 16]. Using intraoperative X-ray, the inclination and anteversion may be measured. This is crucial because in using intraoperative X-rays it is possible to correct the acetabular component orientation more properly intraoperatively.

Widmer’s method can be used to measure the acetabular cup component intraoperatively and to reorient it immediately if necessary [9]. Since this method shows linear correlation in the range of S / TL 0.2-0.6, this ratio can be measured intraoperatively relatively easily. A table suggested by Widmer can be used in the operating room wall.

In this study, we wanted to evaluate the effectiveness of the intraoperative C-arm image intensifier to correct the orientation of the acetabular component with an application of Widmer’s method intraoperatively. The purpose of the present study was twofold. First, we wanted to determine in what percentage of cases a single intraoperative AP hip X-ray would change orientation of the acetabular component. Second, after that, we wanted to determine in what percentage of measurements taken on the postoperative CT scans as reference standard would be in a safe target zone after operation.

## Methods

This research project has been reviewed and approved by the Institutional Review Board (IRB) of the authors’ affiliated institutions.

Consecutive patients who underwent primary total hip arthroplasty (THA) March 2017- August 2018 were retrospectively reviewed. All THAs in this study were performed at one institution by one attending joint arthroplasty surgeon. All hips were replaced through a posterolateral approach with the patient in a lateral decubitus position using Pinnacle acetabular cups (DePuy, Warsaw, IN, USA). These patients were followed up for a minimum of 1 year after surgery.

Inclusion criteria were: 1) All ages; 2) THA performed March 2017-August 2018; 3) Primary THA performed using modified Gibson’s posterolateral approach; 4) DePuy Synthes Pinnacle acetabular component was used; and 5) Radiological and clinical data were available preoperatively, immediate postoperatively, at 3 months and 1 year after surgery. Exclusion criteria were: 1) Follow-up loss; 2) Other combined complications such as periprosthetic joint infection; 3) Cases that other acetabular components were used.

### Operative technique

Preoperative templating was conducted to determined the acetabular component size and proximal femur geometry based on standardized plain radiographs on a picture archiving communication system (PACS) system (Health Tech Solutions, FL). The sizing by the templating facilitated selection of the size of the acetabular component in actual surgery. The target ranges for preoperative, inclination and anteversion of the acetabular component were set at 30-50 degrees and 15-35 degrees, respectively [17].

The surgical procedures were generally the same. Patients were placed in the lateral decubitus position and the same surgeon performed all surgeries through a modified Gibson’s posterolateral approach.

**Fig. 1:**
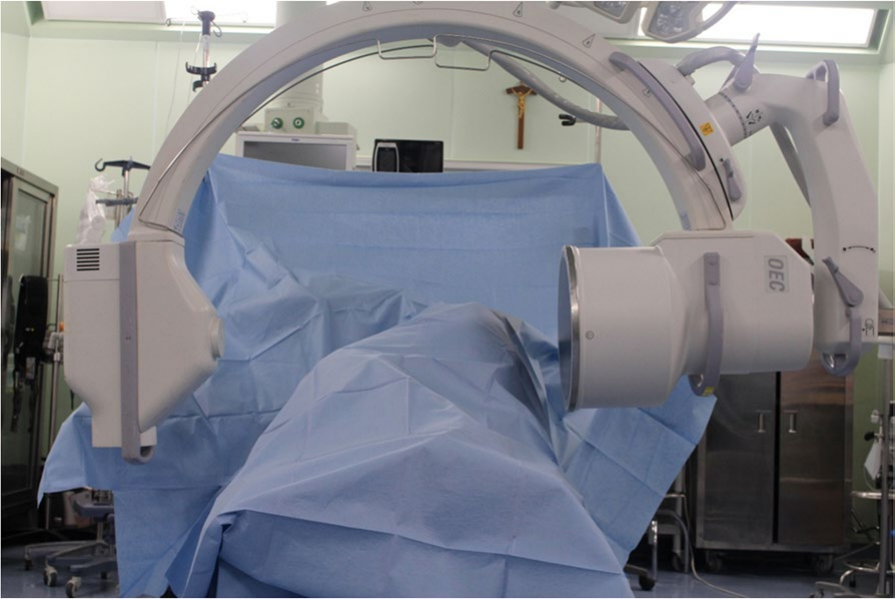
An actual example of an operating room for intraoperative radiography in this hospital. The patient is in a lateral decubitus position. A temporary sterile drape is over the patient. The X-ray by the C-arm intensifier is shot cross-table

**Fig. 2:**
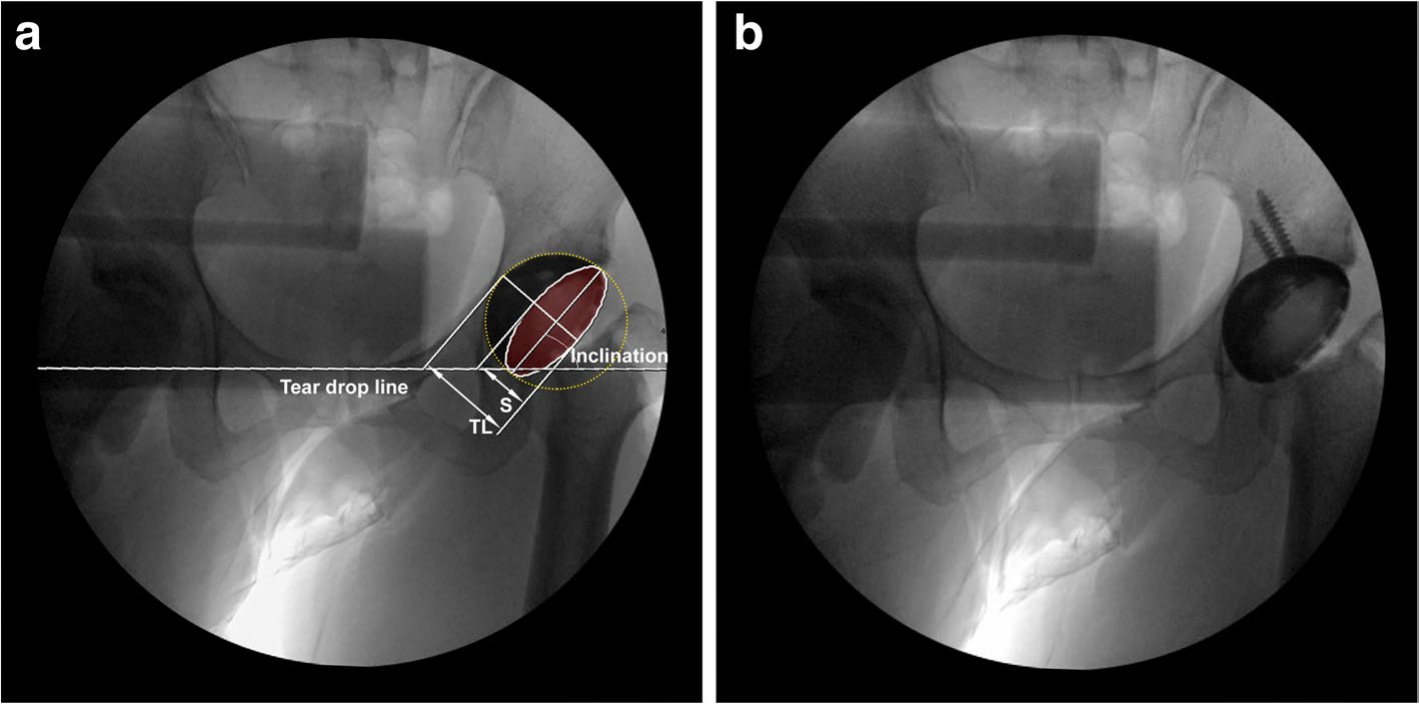
A single anteroposterior (AP) hip X-ray was taken intraoperatively after temporary insertion of acetabular component (**a**). Another anteroposterior (AP) hip X-ray was taken if adjustments were made, the next step, screws insertion and fixation of the acetabular component was proceeded (**b**)

The actual configuration of the operating room for taking radiographs during surgery is shown in Fig. 1. A sterile drape is used to prevent contamination of the patient, and the C-arm is placed horizontally adjacent to the patient’s hip. The anteroposterior (AP) hip radiograph is then taken in a cross-table fashion according to Murray centered on the symphysis and showing both hips [3, 9]. In all cases, an AP hip X-ray was taken intraoperatively after temporary insertion of acetabular component (Fig. 2A) in position in place. This X-ray send to the PACS system directly and evaluated for cup inclination and anteversion using Widmer’s method. The PACS system incorporates digital goniometers and digital rulers for radiographic measurements. This evaluation is also designed to be feasible on the C-arm screen. X-ray was evaluated and, if necessary, the position of the acetabular cup was adjusted. If the adjustments were made the component, another intraoperative AP X-ray was taken and evaluated again. If no change, the next step, which is screws insertion and permanant fixation of the acetabular component was proceeded (Fig. 2B). At the end of the operation, it was recorded whether the orientation of the acetabular cup was changed through X-ray evaluation.

A postoperative X-ray centered on the symphysis was taken for each patient just after the surgery. However, in this study, postoperative X-ray was not used as reference. Instead, the 3D CT scan was used to determine final alignment postoperatively. Postoperative CT scans were obtained at 7 days after THR using a dual-source 128-slice CT scanner and were considered the reference standard. The rate at which the intraoperative X-ray resulted in the acetabular component being corrected during the operation and the percentage of acetabular cup orientations that hit the target zone as measured on the postoperative CT scans was determined.

### Radiologic parameters

The intraoperative AP hip X-rays were acquired using the C-arm image intensifier (GE OEC 9900 Elite C-arm, GE OEC Medical System, UT). Although the range of the C-arm image is limited, the plain radiograph centered on the pubic symphysis displays both hips. Pelvic tilt can be judged by looking at pubic symphysis to sacrococcygeal distance with normal values of 32 mm (range 8-50 mm) in male and 47 mm (range 15-72 mm) in female patients [18]. This distance was estimated to be within the normal range through comparison with the diameter of the acetabular component. This X-ray was sent to the PACS system directly and was used to evaluate cup inclination, and cup anteversion using Widmer’s method. All intraoperative measurements of orientation of the acetabular component by the C-arm image intensifier could be measured by the S/TL ratio without referring to their absolute length. All postoperative radiographs (X-rays) used for this study were digital images viewed on the Marosis m-view PACS system.

The inclination was defined as the angle between the face of the cup and the transverse interteardrop line [3]. Also, the acetabular component anteversion intraoperatively by Widmer’s method was evaluated (Fig. 2A) [9]. The short axis (S) is the distance of the short axis of an ellipse drawn perpendicular to the long axis of the acetabular component. Total length (TL) is the entire distance of the projected cross section of the acetabular component along the short axis. This method shows linear correlation in the range of S/TL 0.2-0.6. The rest of the calculations were reported in a table attached to the operating room [9].

**Fig. 3:**
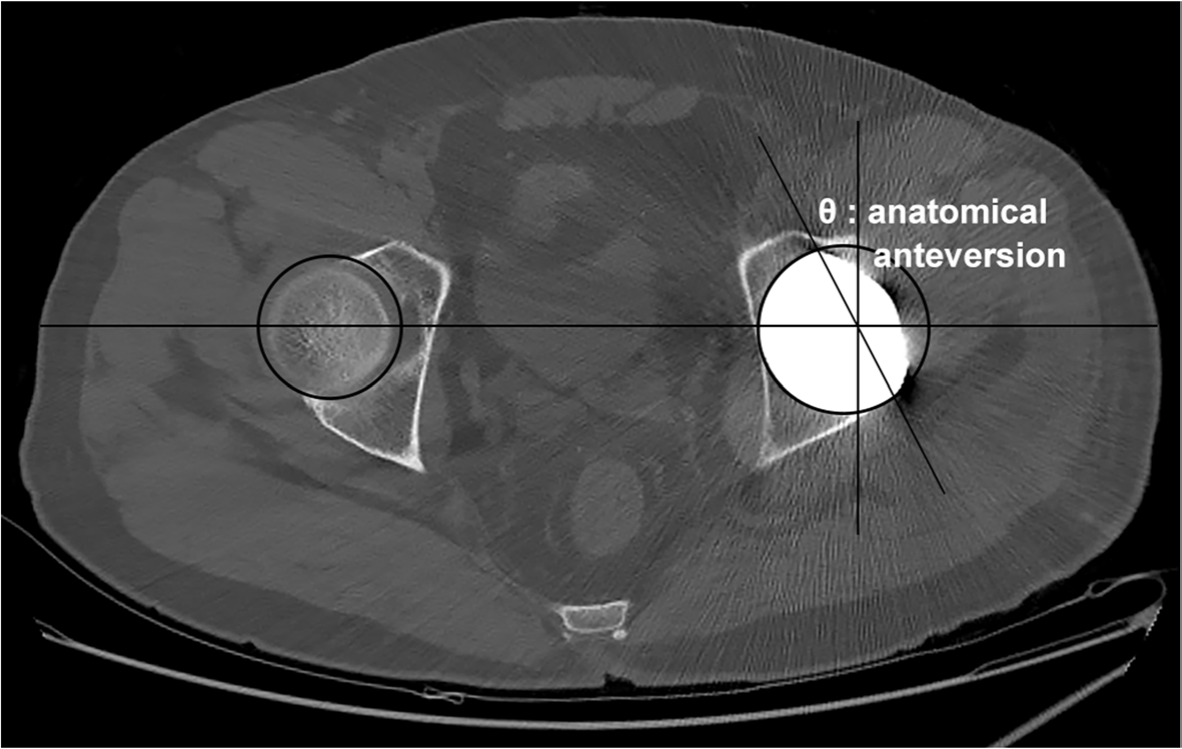
Assessment parameters for measuring anatomical anteversion on CT scans by the method from Murray’s concept

Postoperative CT scans were obtained at 7 days after THA with a dual-source 128-slice CT scanner (Somatom Definition Flash, Siemens Healthcare, Germany) and were regarded as the reference standard. On CT scans, the method from Murray’s concept was used to measure the anatomic acetabular version [3]. In the axial view, the cut showing the largest acetabular component was selected. Circles were drawn along the edge of the implant on the operated side and the acetabulum on the opposite side. Then a line connecting the centers of the two circles, a line perpendicular to it at the center of the circle on the operated side and another oblique line from the most anterior point of the component to its most posterior point were drawn. The anatomical version was measured through the angle between the vertical and tangential lines (Fig. 3).

### Statistical analysis

Two single-blinded reviewers (clinical fellows in hip and pelvis division) evaluated intraoperative X-rays and 3D CT scans without knowing if the intraoperative changes occurred after the surgery. In the cases wherein the components were changed based on the intraoperative X-rays, the final intraoperative X-ray was evaluated. Reproducibility was assessed based on the intraclass correlation coefficient (ICC). Each reviewer measured the inclination and anteversion on intraoperative X-rays and CT, twice 1 week apart. The intra-observer reliability was assessed using the values measured by one examiner. Inter-rater reliability was also measured by comparing the means of two observers. Reliability measurements were reviewed and the results reached ‘almost perfect agreement’. Sample size was calculated using the effect size 0.5, the alpha error accepted was 0.05, and the beta error was 0.2 to ensure power of 80%. The estimation indicated that it would be necessary to include at least 34 cases in the analysis. The paired t-test was used for the comparison of intraoperative X-ray and CT scans measurement. The statistical analysis was performed using the SPSS 20 software (SPSS Inc., Chicago, IL, USA).

## Results

Among a total of 121 consecutive cases, 20 hips were excluded (10 cases were excluded due to follow up loss, other implants were used in eight cases, and two cases were excluded as complications of periprosthetic joint infection), finally 101 cases (91patients, 47 males and 44 females, mean age 61.1 (range 46-84)) were enrolled in this study. Patients demographics and preoperative diagnoses were collected at the history and physical examination, which occurred up to 7 days preoperatively (Table 1). Uncemented fixation was used in 100% of cases.

**Table 1:** Patients demographics and reason for THA

Demographics	N	Mean (SD)	Range
Age	101	61.1(11.3)	46-84
Sex	91 (100%)		
Female / Male	47 / 44 (51.6% / 48.4%)		
Height (cm)	101	162.7(10.1)	130-189
Weight (kg)	101	62.8(10.9)	40-98
BMI	101	23.7(3.1)	17.8-32.0
F/U period (month)	101	23.5(9.5)	12-51
Harris hip score(12 M)	101	94.2(5.9)	82-98
Reason for THA	N	%	
ONFH	62	61.4%	
Primary OA	17	16.8%	
Secondary OA	4	4.0%	
Neck fracture	13	12.9%	
Rheumatoid arthritis	2	2.0%	
LCP/Dysplasia	3	3.0%	

The intraoperative X-ray led to a change in the intraoperative management in 8.9% of cases (Table 2). Among the 8.9% of cases wherein the X-ray changed the management, the changes included an alteration of cup inclination in 2.0% and version in 6.9%.

**Table 2:** Frequency of change in intraoperative management

Frequencies of management	N	%
No	92	91.1%
Yes	9	8.9%
Change in operative plan		
Cup inclination	2	2.0%
Cup anteversion	7	6.9%

In all radiological measurements, the intra-observer and inter-observer reliabilities were acceptable (> 0.90) (Table 3). The mean alignment parameters and standard deviations for the entire cohort are summarized in Table 4. Also, comparison of the final intraoperative X-ray and 3D CT scan are summarized in this table. The paired t-test didn’t show significant differences between final radiographs and CT scans in inclination and version.

**Table 3:** Intraobserver and interobserver reliability of measurements on final intraoperative radiography and CT scan

	Intra-observer reliability		Inter-observer reliability	
	ICC	95% CI	ICC	95% CI
Inclination on X-ray	0.953	0.918 to 0.973	0.908	0.838 to 0.947
Anteversion on X-ray	0.972	0.952 to 0.984	0.966	0.940 to 0.981
Inclination on CT	0.929	0.876 to 0.960	0.915	0.850 to 0.951
Anteversion on CT	0.913	0.847 to 0.950	0.931	0.880 to 0.961

**Table 4:** Final intraoperative X-ray and 3D CT scan evaluation

Variable	Target cup angle (degrees)	N	Mean (SD)	Median	Range	*p*-value
Cup inclination (X-ray)	30-50	101	43.9(3.8)	44.0	35.0-50.0	0.122
Cup inclination (3D CT)	30-50	101	43.7(3.7)	43.0	33.4-49.4	
Cup anteversion (X-ray)	15-35	101	27.5(5.1)	28.7	15.6-34.8	0.068
Cup anteversion (3D CT)	15-35	101	28.0(5.0)	28.2	15.0-34.9	

The impact of the intraoperative X-ray is summarized in Table 5. Approximately 9% of outlying components were identified and put in the target range. In all of the cases, repositioned acetabular cup was placed within the target zone using the intraoperative C-arm image intensifier and CT scans. The mean Harris hip score (HHS) after total hip arthroplasty in 1 year was 94.2 (82-98) in this study group. However, during the follow up after surgery, one patient was hospitalized with repeated dislocations. Although the acetabular component orientation was in target range in this patient, three dislocations occurred three, five and nine weeks after surgery. This patient was female with a history of lumbar fusion and had low activity and abductor muscle weakness. Due to the recurrent dislocations, this patient has been revised with a constrained liner.

**Table 5:** Usefulness of intraoperative radiographs

Variable	Satisfactory on initial intraoperative X-ray	Satisfactory on final intraoperative X-ray	Satisfactory on final 3D CT scan
Cup inclination	99/101(98.0%)	101/101(100.0%)	101/101(100.0%)
Cup anteversion	94/101(93.1%)	101/101(100.0%)	101/101(100.0%)

## Discussion

Hip dislocation is one of the most common complications following total hip arthroplasty (THA). Registry-based studies have reported that dislocation is among the leading causes of revision after primary THA [19, 20]. Factors influencing the risk of dislocation are many and complex. Malposition of the acetabular component is one of the risk factors for dislocation after THA. Various safe ranges are proposed by many authors [2, 4, 17]. However, several investigators have questioned whether the historic concept of a safe zone is clinically relevant [21, 22].

There is an opinion that combined anteversion is more crucial, that incorporates femoral stem and acetabular orientation, may be a better indication, but is considerably more difficult to measure and this may require further investigation. Many combined anteversion patterns by many authors have been reported [23, 24]. However, there are also various theories and consensus not yet reached. Combined version is an additional parameter that helps to optimize the patient-specific positioning of the components in addition to the cup anteversion and inclination [25–27]. Thus, it is probably not sufficient to determine the safe zone by considering only the acetabular component.

Due to the multifactorial nature of THA dislocation, safe zone for cup positioning in THA could not be justified alone. Several patient and surgery-related risk factors for dislocation have been identified, including spinal fusion and stiff spine. According to the nationwide database, a history of spinal fusion was the most significant independent risk factor for early dislocation within 6 months [28]. This is because the pelvic tilt directly affects the acetabular version [18, 29, 30]. To take adequate AP hip X-rays with proper pelvic tilt, uniform AP hip radiographs were taken in a cross-table fashion centered on the superior aspect of the pubic symphysis and perpendicular to the patient. The pubic symphysis to sacrococcygeal distance was within the known normal range in this study. However, the results could be inaccurate when the pelvis is tilted or when the contralateral hip joint or the lumbar spine is stiff [11].

Dislocation after THA is more common in patients with a lumbar spinal fusion [31]. A fused spine stiffens the lumbar segments and directly affects mobility of the pelvis. Spinal fusions to the pelvis may result in a reduction in pelvic tilt in sitting, this may explain why patients with a lumbar spinal fusion have a higher rate of posterior dislocation. In this study, one case of dislocation occurred even though the acetabular component was in the target range in the patient with a lumbar fusion.

Since a modified Gibson’s posterolateral approach was used in this study, the target cup range was set up more anteverted than Lewinnek’s proposal. A posterolateral approach may influence soft tissue and muscular weakness at the surgical site, predisposing to posterior dislocation [2, 17]. Though there are various opinions on the scope of safe zone, and there is no consensus as mentioned above, it is encouraging to set the target range, put the acetabular component in the desired zone, and present the possibility of reducing the rate of dislocations.

Measuring the anteversion of acetabular component is more difficult to measure than inclination. There are several methods and also, other Area and Orthogonal methods have been introduced [32]. However, these methods used to focus on measuring the version after surgery. In this study, Widmer’s method was applied intraoperatively and the results showed that there was no significant difference from the measured value in CT scans as reference.

Assuming the safe zone and whatever its range, there has been minimal research on the proper way to put components into the safe zone intraoperatively. According to the literature, many surgeons tried to set up the safe zone and put the component inside it using X-rays, but without specific methods, it was difficult to put it stably [16]. With an application of Widmer’s method, the acetabular component could be reliably placed in the target range in this study. Whether other methods can be applied during surgery may need more investigation.

The Widmer’s method assumed that radiographic cup orientation is measured on a plain radiograph centered on the symphysis and showing both hips. However, because of the narrow field of view of C-arm used, it is difficult to conduct research in this ideal situation. Although the range of the C-arm image is limited, the intraoperative radiograph centered on the symphysis displays both hips. X-rays focused on the acetabular component have also been investigated, in which this situation can affect the outcome. Further research is needed to determine wherein the results of measurements can vary depending on the focus.

Although controversial, there are reports showing that the reliability of the validity of the Widmer method is not satisfactory. However, this formula was adopted since it was considered the most appropriate method available intraoperatively. There is a desire to be cautious in that we have identified the possibility of changing the acetabular component properly during surgery.

Since the CT scan is known as a more accurate imaging tool to measure the acetabular component, we want to use the postoperative CT scans measurement as reference standard. The resulting costs are not negligible, and the authors’ country may compensate the cost to some extent with the expansion of medical insurance.

This study has several limitations that warrant discussion. First of all, Widmer’s method measures radiographic anteversion and inclination. On the other hand, measurements on CT scans provide the anatomical anteversion on the axial CT slices, whereas it provides the radiographic inclination on coronal CT slices. This distinction is very important because the identical cup position is expressed by different numerical values in the radiographic and in the anatomical definition and it may affect the results and conclusions. Therefore, comparing CT measurements to radiographic measurements on plain radiographs are only correct after transforming them into the same definition, either radiographic or anatomic. Since previous studies have shown no statistical difference between anatomical and various radiological anteversion, this anatomic anteversion from the axial CT slices was used as reference standard in this study [7, 33]. Second, although there were enough cases to assess differences and draw conclusions, the sample size was relatively small, and it was a retrospective study design. Therefore, it cannot be completely free from selection bias and unfortunately, this study didn’t give the robustness of data offered by prospective data collection. Third, since pelvic tilt has a direct influence on the appearance of acetabular component version, efforts were made to set the pelvis to a neutral position by adjusting the distance between sacrococcygeal joint and pubic symphysis. However, due to technical limitations, it was not possible to accurately ensure no pelvic tilt, which may affect results and conclusions. Fourth, Asians are relatively small in size and skeleton, and we are not sure if this study can be applied to a larger population.

The method of Widmer was accurate using intraoperative imaging intensifier for the measurement of the anteversion of the acetabular component during THA, when compared to the anteversion obtained from the 3D computed tomography. Using the intraoperative C-arm imaging was very valuable because it allowed for correction of the position of the acetabular cup intraoperatively. Ultimately, with an application of Widmer’s method frequency of subsequent early dislocations can be approximately 1% which was not above than known figures. The mean Harris hip score after THA in 1 year was 94.2 (82-98).

## Conclusions

The method of Widmer was accurate using intraoperative imaging intensifier for the measurement of the anteversion of the acetabular component during THA, with reference to the anteversion obtained from the 3D computed tomography. Also, using the intraoperative C-arm imaging was very valuable because it allowed correction of the position of the acetabular cup during the surgery.

## Data Availability

The datasets used and/or analyzed during the current study are available from the corresponding author on reasonable request.

## Abbreviations

3D: Three-dimensional
n.s.: Not significant
S/TL: Short axis/Total length
